# The Degradation Pathway of the Mitophagy Receptor Atg32 Is Re-Routed by a Posttranslational Modification

**DOI:** 10.1371/journal.pone.0168518

**Published:** 2016-12-16

**Authors:** Mariia Levchenko, Isotta Lorenzi, Jan Dudek

**Affiliations:** Department of Cellular Biochemistry, Georg-August University, Göttingen, Germany; University of Alabama at Birmingham, UNITED STATES

## Abstract

The outer mitochondrial membrane protein Atg32 is the central receptor for mitophagy, the mitochondria-specific form of autophagy. Atg32 is an unstable protein, and is rapidly degraded under conditions in which mitophagy is not induced. Here we show that Atg32 undergoes a posttranslational modification upon induction of mitophagy. The modification is dependent on the core autophagic machinery, including Atg8, and on the mitophagy-specific adaptor protein Atg11. The modified Atg32 is targeted to the vacuole where it becomes stabilized when vacuolar proteases are deficient. Interestingly, we find that this degradation pathway differs from the degradation pathway of non-modified Atg32, which neither involves vacuolar proteases, nor the proteasome. These analyses reveal that a posttranslational modification discriminates a form of Atg32 targeting mitochondria for mitophagy from that, which escapes mitophagy by rapid degradation.

## Introduction

Mitochondria are essential organelles that fulfill the cellular energy demand by oxidative phosphorylation and play important roles in heme generation, Fe-S cluster biosynthesis and the regulation of apoptosis. Damaged mitochondria are detrimental to the cell and have been implicated in diseases including heart failure, Alzheimer’s disease, Parkinson’s disease and cancer [1,2]. Evolutionary well-preserved quality control mechanisms prevent mitochondrial malfunction and remove damaged or excess mitochondria. Autophagy is a highly regulated process, in which cellular constituents are separated from the cytosol within a double membrane vesicle, the autophagosome [3]. Autophagosomes fuse with the lysosome, where contents are degraded and recycled. Selective forms of autophagy have been shown to clear cellular contents or superfluous or damaged organelles, such as ribosomes (ribophagy), peroxisomes (pexophagy) or the nucleus (PMN, piecemeal microautophagy of the nucleus) [4–6]. Mitophagy is a mitochondria-specific form of autophagy, which plays an important role in removing damaged mitochondria. Mitophagy is induced during transition from exponential growth to the stationary phase in yeast *Saccharomyces cerevisiae*, removing any surplus of mitochondria, and can also be triggered by nitrogen starvation or rapamycin treatment, providing essential nutrients to the starved cells [7,8].

Screens in baker's yeast for genes, essential for the formation of autophagosomes have revealed a set of "core autophagy-related" (Atg) proteins, that are involved in non-selective as well as in selective forms of autophagy including mitophagy [9–11]. The induction of mitophagy is under the control of the Atg1-Atg13 kinase complex, which integrates signals from the protein kinase Tor (Target of rapamycin), involved in the regulation of cell growth in response to changes in nutrients conditions. The activation of the Atg1-Atg13 complex triggers the formation of an isolation membrane at a specific location within the cell, called phagophore assembly site (PAS) [12,13]. For the expansion of this membrane structure, the Phosphatidylinositol-4,5-bisphosphate 3-kinase (PI3K) complex is directed to the PAS with the help of Atg14, where it generates PI3P, thereby establishing the platform for autophagosome biogenesis [14,15]. Two ubiquitin-like conjugation systems control the elongation of the phagophore membrane systems [16]. The conjugation of the ubiquitin-like protein Atg12 to Atg5 is mediated by Atg7 and Atg10 [17]. The formation of an Atg12-Atg5-Atg16 complex then induces the second ligation process, in which the ubiquitin-like Atg8 is cleaved by Atg4 and conjugated with the membrane lipid phosphatidylethanolamine (PE) with the help of Atg7 and Atg3 [18–20]. Membrane-conjugated Atg8 serves as a recruitment site for the autophagic machinery, which targets the autophagosome to the lysosome, or vacuole in yeast [21]. Atg15-mediated lysis of the autophagosomal membrane within the vacuole induces hydrolysis of the cargo by various vacuolar hydrolases, including Pep4 [8,22].

To allow the specific removal of mitochondria, mitophagy-specific receptors are required to label the targeted organelles. In mammals, NIX [23], Bnip3 [24] and FUNDC1 [25] are mitochondria-specific receptor proteins. The only known receptor in yeast, Atg32, has been identified through two independent genome-wide screens for non-essential gene deletion mutants specifically defective in the degradation of mitochondria [26,27]. Yeast lacking the Atg32 protein show mitochondrial defects, like mtDNA damage and increased levels of reactive Oxygen Species (ROS) [28]. Atg32 spans the outer mitochondrial membrane with one predicted single-helical transmembrane (TM) domain, exposing an N-terminal 43 kDa cytosolic domain and a C-terminal 13 kDa mitochondrial Intermembrane Space (IMS) domain [26,27]. A mammalian homologue of yeast Atg32 has been identified, recently [29,30].

The cytosolic domain of yeast Atg32 mediates interaction with several components of the autophagy machinery. The interaction with the adaptor protein Atg11 is enhanced under mitophagy-inducing conditions, like nitrogen starvation [26,27]. The binding of Atg11 to Atg32 recruits the dynamin-related GTPase Dnm1 to mitochondria, promoting mitochondrial fission and segregation [31] and mitochondrial targeting to the PAS [26,27]. This step is regulated by casein kinase 2, phosphorylating Atg32 at Ser114 and Ser119 and thereby promoting interaction of Atg32 and Atg11 [32]. In addition, processing of the Atg32 IMS domain by the inner membrane i-AAA protease Yme1 has also been shown to enhance Atg11 binding [33]. The interaction of Atg32 with Atg8 occurs via the Atg8 family interacting motive (AIM) or LC3 interacting region (LIR), within the cytosolic domain of Atg32 [26,27]. Binding to Atg8 was shown to be dependent on the phosphorylation close to the AIM region (Ser81, Ser83, Ser85) [34]. The mitogen-activated kinases (MAPK) Slt2 and Hog1 have also been reported to be involved in mitophagy, however the kinase directly responsible for Atg32 phosphorylation has not been identified [35]. In addition, when core components of the Ubp3-Bre5 deubiquitination complex were found to dynamically translocate from the cytosol to mitochondria upon induction of mitophagy, a role of ubiquitination in the regulation of mitophagy was suggested [36].

As the induction of *ATG32* gene expression does not coincide with the induction of mitophagy [27], we speculate that additional steps are required for the activation of Atg32. Covalent modification is discussed as a central mechanism for the regulation of Atg32 activity. Here, we demonstrate a novel modification of Atg32, which specifically labels mitochondria destined for rapid degradation in the vacuole. We observe this modification under various mitophagy triggers. We address the involvement of the different key players of the autophagy machinery and show that the modification is dependent on the core autophagic machinery and the specific receptor protein Atg11.

## Materials and Methods

### Yeast strains and growth conditions

*Saccharomyces cerevisiae* deletion strains and ATG32^ZZ^, ^ZZ^ATG32 and ^ZZ^ATG32_IMS_ strains were of the BY4742 background (Euroscarf, Frankfurt, Germany), YPH499 *(MATα ura3-52 lys2-801_amber ade2-101_ochre trp1-Δ63 his3-Δ200 leu2-Δ1*) or derived from WCG4*α* (MAT*α ura3 his3-11*,*15 leu2-3*,*112*) [9]. For the expression of Ubiquitin fused with Hemagglutinine (HA) at its N-terminus under control of the CUP1 promoter the plasmid YEp112 was transformed into yeast [37]. All other strains have been generated by chromosomal integration of a PCR fragment consisting of KANMX6 or NATMX4 or HIS3 marker and are listed in the Table 1 [38,39]. Transformants were selected on minimal media or on plates containing 200 μg/l kanamycin (Roth, Karlsruhe, Germany) or 100 μg/l nourseothricin (Werner Bioagents, Jena, Germany). Yeast cells were grown in YPD (1% yeast extract, 2% peptone, 2% glucose), YPL (1% yeast extract, 2% peptone, 2% lactate), YPGal (1% yeast extract, 2% peptone, 2% galactose) or YPGlycerol (1% yeast extract, 2% peptone, 2% glycerol). Alternatively, synthetic minimal medium (0.67% yeast nitrogen base, amino acids, and vitamins), containing 2% glucose (SD) or 2% lactate (SL) was used. Mitophagy was induced using 0.2 μg/ml rapamycin or by switching to synthetic minimal medium lacking nitrogen (SD-N; 0.17% yeast nitrogen base without amino acids, 2% glucose).

**Table 1:** Yeast strains used in this study.

Name	Genotype	Reference
BY4741	MATa *his3Δ1; leu2Δ0; met15Δ0; ura3Δ0*	Euroscarf
*atg32Δ*	BY4741 *atg32Δ*::*kanMX4*	Euroscarf
Atg32^ZZ^	YPH499 atg32::ATG32-TEV-ProA-His7-HIS3	This study
^ZZ^Atg32	YPH499 atg32::HIS3-NOP1pr-His7-ProA-TEV-ATG32	This study
^ZZ^Atg32_IMS_	YPH499 atg32::HIS3-NOP1pr-His7-ProA-TEV-ATG32(382–529)	This study
^ZZ^Atg32	BY4741 *atg32*::*HIS3-NOP1pr*-His7-ProA-TEV-*ATG32*	This study
^ZZ^Atg32 *pep4Δ*	BY4741 *pep4Δ*::*kanMX4*; *atg32*::*HIS3-NOP1pr*-His7-ProA-TEV-*ATG32*	This study
^ZZ^Atg32 *pep4Δ atg1Δ*	BY4741 *pep4Δ*::*natMX6*; *atg1Δ*::*kanMX4*; *atg32*::*HIS3-NOP1pr*-His7-ProA-TEV- *ATG32*	This study
^ZZ^Atg32 pep4*Δ* atg3*Δ*	BY4741 *pep4Δ*::natMX6; *atg3Δ*::*kanMX4*; *atg32*::*HIS3-NOP1pr*-His7-ProA-TEV- *ATG32*	This study
^ZZ^Atg32 *pep4Δ atg4Δ*	BY4741 *pep4Δ*::natMX6; *atg4Δ*::*kanMX4*; *atg32*::*HIS3-NOP1pr*-His7-ProA-TEV- *ATG32*	This study
^ZZ^Atg32 *pep4Δ atg5Δ*	BY4741 *pep4Δ*::natMX6; *atg5Δ*::*kanMX4*; *atg32*::*HIS3-NOP1pr*-His7-ProA-TEV- *ATG32*	This study
^*ZZ*^Atg32 *pep4Δ atg7Δ*	BY4741 *pep4Δ*::natMX6; *atg7Δ*::*kanMX4*; *atg32*::*HIS3-NOP1pr*-His7-ProA-TEV- *ATG32*	This study
^ZZ^Atg32 *pep4Δ atg8Δ*	BY4741 *pep4Δ*::natMX6; *atg8Δ*::*kanMX4*; *atg32*::*HIS3-NOP1pr*-His7-ProA-TEV- *ATG32*	This study
^ZZ^Atg32 *pep4Δ atg10Δ*	BY4741 *pep4Δ*::natMX6; *atg10Δ*::*kanMX4*; *atg32*::*HIS3-NOP1pr*-His7-ProA-TEV- *ATG32*	This study
^ZZ^Atg32 *pep4Δ atg12Δ*	BY4741 *pep4Δ*::natMX6; *atg12Δ*::*kanMX4*; *atg32*::*HIS3-NOP1pr*-His7-ProA-TEV- *ATG32*	This study
^ZZ^Atg32 *pep4Δ atg13Δ*	BY4741 *pep4Δ*::natMX6; *atg13Δ*::*kanMX4*; *atg32*::*HIS3-NOP1pr*-His7-ProA-TEV- *ATG32*	This study
^ZZ^Atg32 *pep4Δ atg14Δ*	BY4741 *pep4Δ*::*natMX6*; *atg14Δ*::*kanMX4*; *atg32*::*HIS3-NOP1pr*-His7-ProA-TEV- *ATG32*	This study
^ZZ^Atg32*pep4Δ atg15Δ*	BY4741 *pep4Δ*::*natMX6*; *atg15Δ*::*kanMX4; atg32*::*HIS3-NOP1pr*-His7-ProA-TEV- *ATG32*	This study
^ZZ^Atg32 *pep4Δ atg16Δ*	BY4741 *pep4Δ*::*natMX6*; *atg16Δ*::*kanMX4*; *atg32*::*HIS3-NOP1pr*-His7-ProA-TEV- *ATG32*	This study
^ZZ^Atg32_IMS_	BY4741 *atg32*::*HIS3-NOP1pr*-His7-ProA-TEV-*ATG32*(382–529)	This study
^ZZ^Atg32_IMS_ *pep4Δ*	BY4741 *pep4Δ*::*kanMX4*; *atg32*::*HIS3-NOP1pr*-His7-ProA-TEV-*ATG32*(382–529)	This study
^ZZ^Atg32_IMS_ *pep4Δatg11Δ*	BY4741 *pep4Δ*::*kanMX4*; *atg11Δ*::*natMX6*; *atg32*::*HIS3-NOP1pr*-His7-ProA-TEV- *ATG32*(382–529)	This study
WCG4a	MATα *his3-11; 15 leu2-3; 112 ura3*	Hilt et al., 1993
^ZZ^Atg32	WCG4a *atg32*::*HIS3-NOP1pr-His7-ProA-TEV-ATG32*	This study
^ZZ^Atg32 *pep4Δ*	WCG4a pep4*Δ*::kanMX4; atg32::HIS3-NOP1pr-His7-ProA-TEV-ATG32	This study
^ZZ^Atg32 *atg11Δ*	WCG4a *atg11Δ*::*natMX6*; *atg32*::*HIS3-NOP1pr*-His7-ProA-TEV-*ATG32*	This study
^ZZ^Atg32 *pep4Δ atg11Δ*	WCG4a *pep4Δ*::*kanMX4*; *atg11Δ*::*natMX6*; *atg32*::*HIS3-NOP1pr*-His7-ProA-TEV- *ATG32*	This study
^ZZ^Atg32 *pre1-1 pre2-2*	WCG4a *pre1-1*; *pre2-2*; *atg32*::*HIS3-NOP1pr*-His7-ProA-TEV-ATG32	This study

### Mitophagy assay

Mitophagy was monitored essentially as described in [40]. In brief, cells used in this assay were transformed with a plasmid encoding for the DHFR-GFP fusion protein, which is targeted to mitochondria by the Su9 presequence (Su9, subunit of the *Neurospora crassa* F_1_F_O_ ATPase). The cells were cultured to stationary phase using selection medium (0.67% Yeast Nitrogen Base w/o amino acids, 0.2% Dropout-Mix, pH 5.5) lacking methionine and supplemented with 2% lactate as sole carbon source. To induce mitophagy, cells were then shifted to starvation medium (SD-N) or treated with 0.2 μg/ml rapamycin and samples were taken at defined time points. Cell extracts were prepared by alkaline lysis and precipitated with trichloroacetic acid. Extracts were separated by SDS-PAGE containing 6 M urea followed by Western blotting.

### Protein isolation

Yeast strains were grown under standard conditions and harvested by centrifugation. Cell lysis was performed by cryogenic grinding using the Retsch MM 301 Mixer Mill (Retsch, Newtown, PA). Grinding was performed in five steps of 3min at 30 Hz and cell powder was resuspended in solubilization buffer (20 mM Tris, 15 mM NaCl 10% Glycerol, 5 mM PMSF, 5 μM pepstatin, 5 mM EDTA, and Roche complete protease inhibitor tablets, pH 7.4). After several clearing steps, cellular membranes were harvested by centrifugation at 16000 g for 10 min. Membranes were solubilized in 1% Digitonin in solubilization buffer. For protein isolation via ZZ-Tag, IgG-chromatography was performed as described in [41,42]. Cellular membranes were solubilized in solubilization buffer (30 mM Tris/HCl, pH 7.4, 80 mM KCl, 10% glycerol, 5 mM MgCl2, and 1% digitonin) at 4°C and subjected to IgG-Sepharose after a clarifying spin. Loaded IgG-Sepharose was washed with solubilization buffer containing 0.3% digitonin and bound proteins were eluted with SDS sample buffer and analyzed by SDS-PAGE and Western blotting.

### HA affinity chromatography

Yeast expressing HA-Ub were homogenized using a beat beater and then solubilized in solubilizing buffer containing in 50 mM Tris, 50 mM NaCl, 10% Glycerol, 1% Triton X-100, pH 7.4 for 30 min at 4°C. Detergent was diluted to 0.1% Triton and solubilized material was clarified by centrifugation at 20.000 g and 4°C for 10 min. Supernatant was loaded onto Monoclonal Anti-HA-Agarose (Sigma, A2095) for 2 h at 4°C. The agarose was washed 10 times with solubilizing buffer containing 0.1% Triton X-100. Bound proteins were eluted with 4mg/ml HA peptide (Roche, 11666975001) and subsequently analyzed by SDS gel and western blotting using HA specific antibody (Roche, clone 12CA5, 11583816001).

### Miscellaneous

For mitochondrial isolation we adapted the established protocol for a reduced procedure time [43]. Yeast cells, equivalent to OD_600_ = 25, were homogenized in a motor driven potter after removal of the cell wall by subsequent treatment with 10 mM DTT and 2 mg/ml Zymolyase (Gerbu, 07663–91). Mitochondria were isolated by differential centrifugation and harvested at 14.000 rpm for 10 min. For deubiquitination of modified Atg32 yeast cell extracts were treated with 4 μM USP2 (BostonBiochem, E-506) in Deubiquitination buffer (50 mM Tris-HCl, 50 mM NaCl, pH 7.4) or 10 mM NEM (N-etylmaleimide, Sigma-Aldrich, 128287) as a control and incubated for 30 minutes at room temperature. Phosohatase treatment was performed by treating cell extracts with 2U alkaline Phosphatase (Roche, 10713023001) in Phosphatase buffer (50 mM Tris, 100 mM NaCl, 10 mM MgCl_2_, 1 mM DTT, 0.5% Triton X-100, 1mM PMSF, pH 8.0) and samples were incubated for 1h at 37°C. 2mM Na_3_VO_4_ was used as a phosphatase inhibitor for controls. For SDS-PAGE and Western blotting of proteins to polyvinylidene difluoride (PVDF) membranes standard techniques were used. Proteins were detected using primary antibodies raised in rabbits and secondary antibodies coupled to horseradish peroxidase (HRP). For detecting GFP a commercial antibody (Roche, Mannheim, Germany) was used. Ubiquitinated proteins were detected using the anti-ubiquitin antibody P4D1 (Santa Cruz Biotechnology, sc8017), for yeast Pdk1 an antibody from Life technologies (459250, clone 22C5D8) was used. The ZZ tag was detected using an peroxidase anti peroxidase (anti-PAP) antibody (HRP-anti-HRP). Signals were detected using a chemiluminescence (ECL) detection system (GE Healthcare) and X-ray films.

## Results

### Enriched fraction of Atg32 reveals protein modification

**Fig 1:**
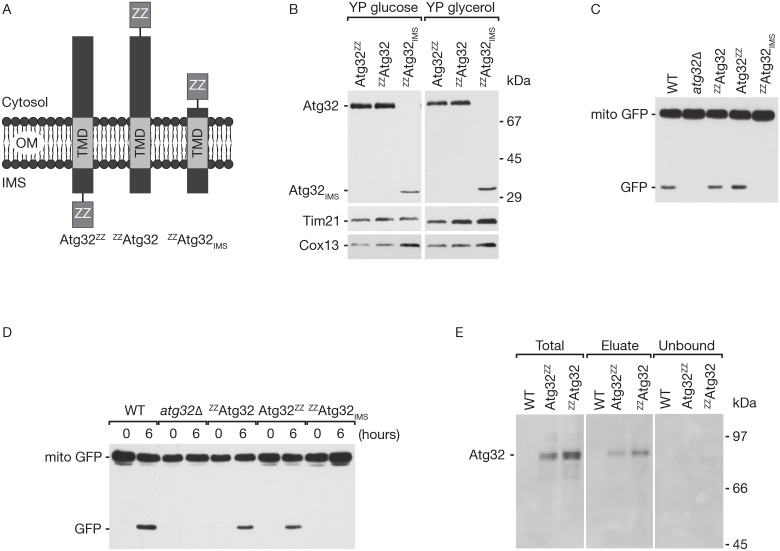
Yeast expressing Atg32 constructs for IgG affinity chromatography. (A) Topologies of Atg32^ZZ^, ^ZZ^Atg32 and ^ZZ^Atg32_IMS_ constructs. ZZ, two repeats of the IgG-binding domain of protein A (Z domain), TMD, transmembrane domain, OM, outer membrane, IMS, intermembrane space. (B) Expression of Atg32^ZZ^, ^ZZ^Atg32 and ^ZZ^Atg32_IMS_ constructs. Lysates of yeast cells grown in indicated media were analyzed by Western blotting with antibodies against the IgG-binding domain (α-PAP) and mitochondrial proteins Tim21 and Cox13, as a control. (C) Analysis of mitochondrial turnover (mitophagy assay). Mito-GFP (DHFR-GFP fused to the presequence of *N*. *crassa* F_1_F_O_ ATPase subunit 9) was expressed in the respective yeast strains and mitophagy was induced by starvation in nitrogen-free media for 6h. Cell lysates were analyzed by Western blotting with antibodies directed against GFP. (D) Mitophagy assay as in (C) after induction of mitophagy by rapamycin treatment (1 μg/ml) for the indicated time points. (E) Total, unbound, and eluate fractions of IgG affinity chromatography using the indicated yeast strains were analyzed by Western blot using the α-PAP antibody.

Atg32 is the central receptor for the specific removal of mitochondria by autophagy in yeast. Upon induction of mitophagy Atg32 is activated and targets mitochondria for their degradation. To elucidate the precise regulation of the activation step of Atg32, we expressed Atg32 fused with affinity tags consisting of two repeats of the IgG-binding domain of protein A (Z-domain) followed by a polyhistidine tag. A TEV cleavage site between Atg32 and the first Z-domain allows for the specific cleavage of the tag by TEV protease treatment. Atg32 is an integral protein in the outer mitochondrial membrane with its N-terminus facing the cytosol and the C-terminus facing the IMS. We designed three different constructs, among which the Atg32^ZZ^ construct exposes the IgG binding site into the IMS, the ^ZZ^Atg32 construct exposes the IgG binding site to the cytosol and the ^ZZ^Atg32_IMS_ construct has the cytosolic domain replaced by the IgG binding domain (Fig 1A). We created yeast strains by integrating corresponding constructs into the endogenous locus of *ATG32* (Table 1). The amount of expression of these constructs was tested by Western blot analysis of yeast cell extracts from the three strains. The ZZ tag was detected using peroxidase anti peroxidase (α-PAP) antibody (HRP-anti-HRP), revealing proteins of the predicted size. The ^ZZ^Atg32_IMS_ construct was detected in reduced amounts compared to the full-length constructs indicating a reduced gene expression or slightly increased protein turnover (Fig 1B).

As Atg32 is essential for mitophagy, we tested the ability of our constructs to induce mitophagy when cells were grown in starvation medium. We therefore expressed mitochondria- localized GFP protein (mito-GFP) consisting of the presequence of *Neurospora crassa* F_1_F_O_ ATPase subunit 9 fused to dehydrofolate reductase (DHFR) and green fluorescent protein (GFP) in the respective yeast strains [40,44]. Upon induction of mitophagy mitochondria are transported to the vacuole for degradation. Proteolysis of mito-GFP in the vacuole results in the formation of a proteolysis-resistant GFP fragment, which allows us to correlate the formation of free GFP with the mitophagy flux. We measured the formation of free GFP by Western blotting after shifting the cells to SD-(N), a nitrogen-free glucose-containing medium, for 6h. Nitrogen starvation caused a robust processing of mito-GFP to produce free GFP. The formation of free GFP is dependent on Atg32, as it is not present in an *atg32Δ* strain (Fig 1C). Expression of ^ZZ^Atg32 and Atg32^ZZ^ allowed for the induction of mitophagy at rates similar to wild-type, whereas the deletion of the cytosolic domain blocked mitophagy induction, in line with previously published results [27]. We also tested mitophagy induction by blocking the TOR kinase with the help of the TOR-specific inhibitor rapamycin [45,46]. Rapamycin-induced mitophagy leads to an efficient generation of free GFP after 6 hours in cells expressing the ^ZZ^Atg32 and Atg32^ZZ^ constructs, but not in cells expressing ^ZZ^Atg32_IMS_, which lacks the cytosolic domain. In summary, N- or C-terminal fusion with ZZ tag did not interfere with mitophagy, whereas truncations of the cytosolic domain did (Fig 1D).

For a biochemical characterization of potential interaction partners, playing a role in Atg32 activation, we used the functional receptor constructs ^ZZ^Atg32 and Atg32^ZZ^ for affinity isolation. In order to avoid protein degradation we developed a protocol to enrich ^ZZ^Atg32 and Atg32^ZZ^ via IgG isolation, while minimizing the extent of proteolysis. Cells were snap-frozen in liquid nitrogen, immediately after harvesting from liquid culture. The frozen pellet was pulverized using a cryo-grinding mill and cellular membranes were solubilized in the presence of the mild detergent digitonin. IgG isolation of the ZZ-tagged protein was performed under protease inhibition and isolated ^ZZ^Atg32 and Atg32^ZZ^ were analyzed by Western blotting (Fig 1E). Both constructs were recovered in the elution fraction. Despite their low binding to IgG Sepharose, the constructs are not detected in the remaining unbound fraction. We therefore speculate that Atg32 is a very unstable protein that is quickly degraded during the isolation procedure, despite the presence of protease inhibitors. The instability of functional Atg32 constructs prevented efficient isolation of interaction partners.

### Atg32 modification directs mitochondria to vacuolar degradation

During the progression of mitophagy mitochondria are delivered to the vacuole, where mitochondrial proteins are degraded by proteases resident to this organelle. We speculate that the degradation of Atg32 in the vacuole in part explains its observed instability. We therefore used a yeast mutant strain to block the turnover of mitochondria in the vacuole. The *PEP4* gene encodes for the proteinase A, a major hydrolase of the yeast vacuole, responsible for maturation of other vacuolar proteases and required for protein turnover during mitophagy [47] [48] [49]. We generated a yeast strain expressing ^ZZ^Atg32 in which *PEP4* was deleted (Table 1). The cells were starved in medium lacking nitrogen to induce mitophagy. We analyzed the protein turnover of ^ZZ^Atg32 by Western blot analysis of cell extracts. Within 2h of starvation the levels of Atg32 have significantly reduced compared to the 0h time point. The turnover of Atg32 is reduced upon deletion of *PEP4*. Interestingly, we found a modified form of Atg32 with a molecular weight of about 100 kDa, which only occurs after mitophagy induction and only in the *PEP4* deletion strain. We concluded that Atg32 undergoes a posttranslational modification after mitophagy induction, increasing the size of the protein by about 20 kDa. The modified form, which is turned over rapidly in wild-type cells, is specifically stabilized by the deletion of Pep4.

**Fig 2:**
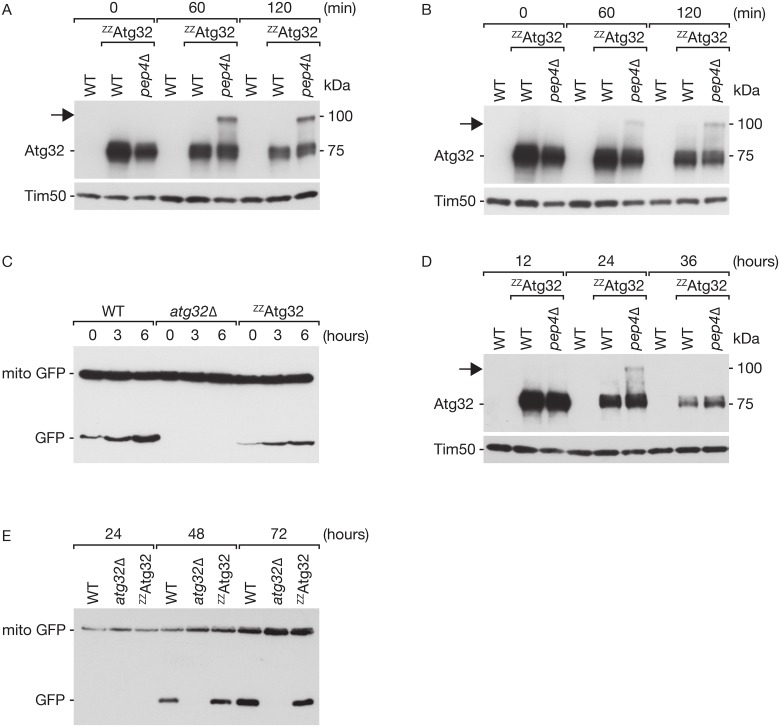
Blocking vacuolar degradation stabilizes modified Atg32 upon mitophagy induction. (A) Yeast cells expressing ^ZZ^Atg32 were grown in medium lacking any nitrogen source for the indicated time points. Cells were harvested and proteins were analyzed by Western blotting after SDS gel electrophoresis using the α-PAP antibody to detect Atg32, with Tim50 as a control. Arrow indicates the modified form of Atg32. (B) Analysis of Atg32 modification as in (A) after induction of mitophagy using 1 μg/ml rapamycin for indicated time points. (C) Mitophagy assay as shown in (Fig 1C) after indicated time points of rapamycin treatment. (D) Atg32 modification in post log cell lysates, analyzed as in (Fig 2A). (E) Mitophagy assay in cells grown until post log phase for the indicated time points.

We next asked if the occurrence of an Atg32 modification is restricted to nitrogen starvation as a trigger for mitophagy, and therefore also tested other mitophagy inducers. Rapamycin is an inhibitor of the TOR kinase and an efficient inducer of mitophagy [50,51]. When cell lysates were analyzed 1h and 2h after Rapamycin administration, a slower migrating form of Atg32 at 100 kDa was observed in yeast strains, in which *PEP4* was deleted (Fig 2B). Induction of mitophagy upon rapamycin treatment was verified by the accumulation of free GFP processed from mito-GFP. Wild-type, but not *ATG32*-deficient cells, showed robust GFP-processing, which was also mediated by ^ZZ^Atg32, albeit with a slower kinetic compared to the wild-type (Fig 2C). When cells were cultivated in full growth medium (YPL), mitochondrial turnover was initiated in post log phase. We detected a modified form of Atg32 at 100 kDa also in post log conditions after 24 h. Degradation of mitochondria continued after 24h, so that Atg32 amounts were strongly reduced below amounts enabling the detection of the modified form. We monitored the extend of mitochondrial degradation for cells growing for 24h, 48h and 72h in YPL using the GFP-based mitophagy assay. A robust induction of mitophagy was not only observed in wild-type cells but also in the ^ZZ^Atg32 strain (Fig 2E).

In order to confirm that the observed modification of Atg32 occurs on mitochondria, yeast cell lysates were separated into a mitochondrial and a cytosolic fraction. We observed that the mitochondrial marker protein Tom40 is enriched, and the cytosolic marker Pgk1 is excluded from the mitochondrial fraction (S1 Fig). On mitochondria non-modified Atg32 was heavily degraded during the procedure. Interestingly, the modified form of Atg32 was exclusively found in the mitochondrial fraction, but not in the cytosol (S1 Fig). This confirms, that Atg32 modification occurs on mitochondria.

### The autophagy core machinery is required for Atg32 modification

**Fig 3:**
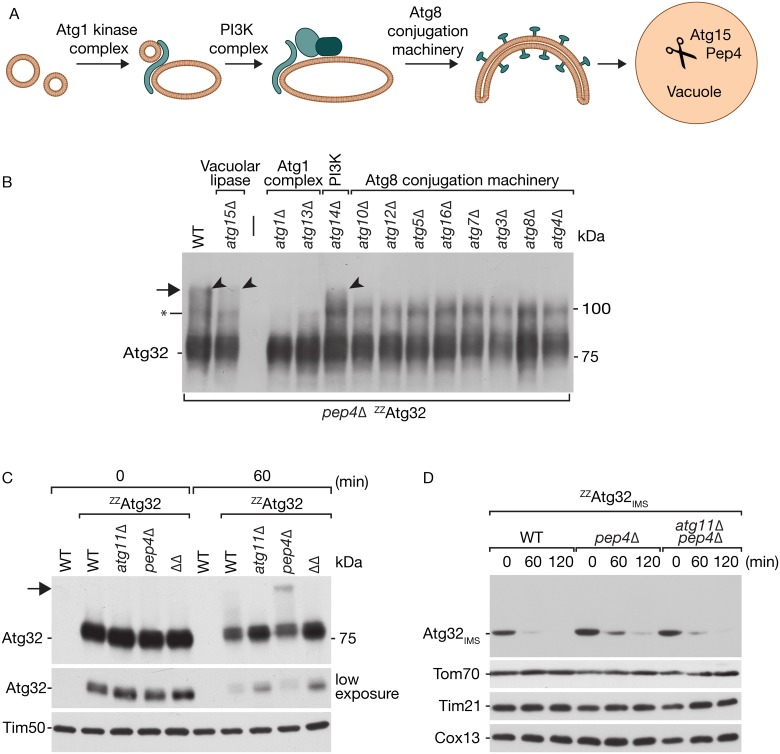
Autophagy-specific genes are required for Atg32 modification. (A) Schematic representation of the sequence of events involved in mitophagy. (B) *pep4Δ* strains expressing ^ZZ^Atg32 and carrying an additional deletion in the indicated gene were induced for mitophagy by 1 μg/ml rapamycin treatment. Cell lysates were analyzed by SDS PAGE and immunoblotting with the α-PAP antibody. Arrows indicate modification products. Asterisk indicates a second modification of Atg32. (C) Indicated yeast strains expressing the ^ZZ^Atg32 construct were treated with 1 μg/ml rapamycin for 0 or 60 min and analyzed by Western blotting using the α-PAP antibody. WT–wild-type, *ΔΔ –*double deletion mutant *atg11Δ pep4Δ*. Arrow indicates a modification product. (D) Experiment as in (C) using cells expressing the ^ZZ^Atg32_IMS_ construct.

Mitophagy is a sequential process, initiated by the activation of the Atg1-Atg13 kinase complex followed by the activation of the phosphatidylinositol 3-kinase (PI3K, Atg14) complex at the PAS [52]. The Atg8 conjugation machinery promotes lipidation of Atg8, which is required for cargo recognition during selective autophagy [52]. Autophagosomes fuse with the vacuole, where lipid membranes are broken down by the lipase Atg15 and cargo proteins are hydrolyzed by proteases activated by Pep4 (Fig 3A). In order to identify the mechanisms leading to the Atg32 modification, we screened through a set of yeast strains carrying deletion mutations within core autophagy genes. We therefore generated gene deletions in the *pep4Δ* yeast strain expressing ^ZZ^Atg32. After mitophagy induction by rapamycin treatment for 2h, cell lysates were analyzed by Western blotting for the modified form of Atg32. The majority of *ATG* genes were essential for the Atg32 modification to occur, confirming the involvement of autophagy in the modification of Atg32. The modification depends on the majority of the early core autophagy genes, and also the cargo-specific receptor Atg8 is required, suggesting that the modification of Atg32 occurs after cargo recognition. As the modification remains stable in the absence of the vacuolar lipase Atg15, we concluded that the modification occurs before vacuolar fusion. Interestingly, the modification was still observed in the absence of Atg14, a central regulator of the phosphatidylinositol 3-kinase activity (Fig 3B).

**Fig 4:**
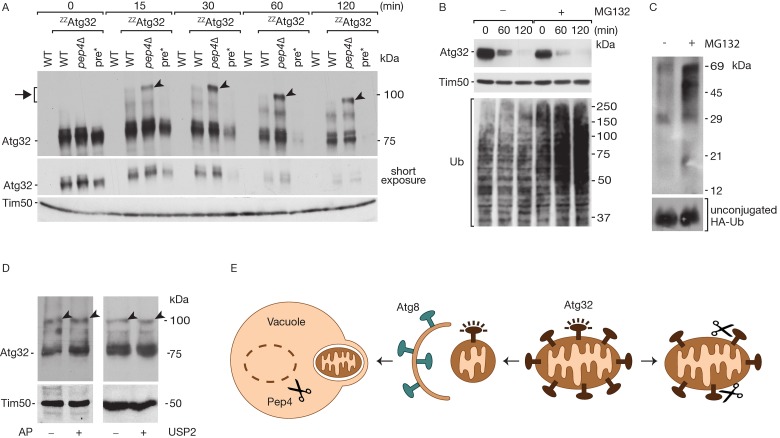
Atg32 modification and degradation is not affected by the defects in the proteasome. (A) Yeast expressing the ^ZZ^Atg32 construct and the indicated mutation in a degradation pathway were analyzed by Western blot after treatment with 1 μg/ml rapamycin for the indicated time periods. Immunoblotting for Atg32 with the α-PAP antibody. WT–wild-type, pre*–pre1-1 pre2-2 mutant. Arrows indicate modification products. (B) The ^ZZ^Atg32 degradation upon mitophagy induction by rapamycin was analyzed in the presence and absence of MG132. Accumulation of ubiquitinated proteins upon administration of MG132 was documented using an ubiquitin specific antibody. (C) Yeast cells expressing HA-Ubiquitin were treated with MG132 or with DMSO as a control. Cell extracts were submitted to HA-affinity isolation and eluates were analyzed by western blot using an HA specific antibody. Unconjugated HA-Ub in the low molecular range was detected as a control. (D) Mitophagy was induced by Rapamycin treatment and cell extracts were treated with alkaline Phosphatase and phosphatase inhibitor as a control (left panel). Cell extracts were treated with the deubiquitinase USP2 or treated with the inhibitor NEM as a control (right panel). (E) Model of Atg32 modification.

We also observed a smaller modified form of Atg32, indicated with an asterisk in Fig 3B. This form might represent a second form of Atg32 modification, however, it also appeared in absence of all *ATG* genes, except for the deletion of *ATG1*. Further analysis showed that the modification occurs independent of the deletion of the vacuolar protease Pep4 (Fig 4A), indicating that this form is independent of the vacuolar degradation pathway. We therefore decided, not to pursue on this modification as it exceeds the scope of our study.

As Atg11 is an essential adaptor protein for selective autophagy, we also included an Atg11-deficient mutant in our study. SDS-PAGE and Western blotting of the whole cell extracts after rapamycin treatment were analyzed for Atg32 modification. The 100 kDa band of Atg32 was readily detected in the *PEP4* deletion strain, but additional deletion of *ATG11* prevented Atg32 modification (Fig 3C). We concluded that a specific block in mitophagy prevents Atg32 modification.

We became interested if the cytosolic domain of Atg32 is required for the modification to occur. Therefore, we expressed the ZZ-tagged Atg32_IMS_ construct in the *pep4Δ* and *pep4Δ*, *atg11Δ* strains and analyzed whole cell extracts after mitophagy induction with rapamycin. No modification of Atg32 was found, suggesting that the modification takes place on the cytosolic domain of Atg32, or that the cytosolic domain is required for the modification to occur. The deletion of *PEP4* also did not stabilize ^ZZ^Atg32_IMS_ as the protein is rapidly degraded below detection by Western blot (Fig 3D). This finding is remarkable as this construct does not support the induction of mitophagy, as shown in Fig 1D. This indicates, that a second pathway, besides mitophagy is responsible for the rapid degradation of non-modified Atg32.

### Mitochondria carrying non-modified Atg32 are excluded from vacuolar degradation

The stabilization of modified Atg32 in a *PEP4* deletion strain identifies the vacuole as its major degradation site upon induction of mitophagy. Interestingly, the degradation of unmodified Atg32 continues, as shown in Fig 2A, 2B and 2D, indicating a separate route of degradation for non-modified Atg32. We therefore speculated on the involvement of the proteasome in the degradation of non-modified Atg32. In order to test this, we expressed ^ZZ^Atg32 in a yeast double mutant carrying a mutation in the subunits Pre1 and Pre2 inhibiting the activity of the proteasome (*pre1-1 pre2-2*; [53,54]). An accumulation of modified Atg32 was not found in proteasome deficient yeast upon induction of mitophagy by rapamycin (Fig 4A). We compared the degradation of Atg32 in proteasome deficient and *PEP4* deficient cells at different time points in this experiment. In *PEP4* deficient cells, the modified form of Atg32 accumulated and unmodified Atg32 was degraded. Surprisingly, we found that non-modified Atg32 was also completely degraded in the *pre1-1 pre2-2* mutant strain. Atg32 was degraded within the first hour of mitophagy induction, indicating an accelerated turnover compared to wild-type and the *pep4Δ* strain (Fig 4A). The accelerated degradation can be explained by an increased autophagy rate in proteasomal mutants, as it has been described before [55].

This surprising finding argues against a role of the proteasome in the degradation of non-modified Atg32. In order to further confirm that the proteasome does not play a role in the turnover of Atg32, we treated cells expressing ^ZZ^Atg32 with the proteasomal inhibitor MG132. After mitophagy induction using rapamycin ^ZZ^Atg32 was still degraded in MG132-treated cells (Fig 4B). Proteasome inhibition was verified by detecting the accumulation of ubiquitinated proteins over time after MG132 treatment using an ubiquitin specific antibody. To provide further evidence that the proteasome is inhibited under our conditions, we used a yeast strain expressing Ubiquitin fused to Hemagglutinine (HA) at its N-terminus [37]. HA-affinity isolation allowed the isolation of HA-Ub conjugated proteins, which were analyzed by western blotting using a HA-specific antibody. MG132 significantly increased the amount of HA-Ub conjugated proteins, confirming a block in proteasomal degradation.

Phosphorylation of Atg32 has been described in the literature as an important regulator of Atg32 function upon mitophagy induction [32–34]. We considered the observed modification of Atg32 to be phosphorylation. We therefore tested if alkaline phosphatase treatment eliminates the modification. However, Atg32 modification resisted alkaline phosphatase treatment (Fig 4C). We also analyzed ubiquitination as a possible mechanism for forming the modification. Treatment with the deubiquitiase USP2 had no effect on the modified form. In order to confirm the activity of USP2, we used HA affinity isolation of Ubiquitin conjugated proteins from MG132 treated yeast expressing the HA-Ub construct. The activity of the enzyme was confirmed by elimination of HA-Ub conjugates in the eluate (data not shown). These data suggest that neither phosphoryation nor ubiquitination are responsible for the formation of the modification.

## Discussion

Mitophagy is a selective degradation of defective and superfluous mitochondria in the cell. The mitophagy receptor Atg32 plays a key role in the quality control mechanism, as it allows to specifically label damaged or superfluous mitochondria [26,27]. Mitochondria once labeled with Atg32 are immediately removed by mitophagy. We have experienced a rapid turnover of Atg32 in our studies, which is explained in part by the fast autophagic removal of Atg32-containing mitochondria. When compared with Tim50, a protein integrated in the inner mitochondrial membrane, Atg32 appears to be degraded with a much faster kinetics. This indicates that not the entire mitochondrial pool, but only damaged mitotchondria labeled with Atg32, are removed. Constant remodeling of the mitochondrial network allows the cell to separate damaged mitochondria from the pool of healthy mitochondria. The mitochondrial pool is a highly dynamic network, in which two opposing processes, fusion and fission shape mitochondrial morphology [56,57]. Fusion allows merging of two mitochondria, whereas fission allows for segregating mitochondria. This principle is important for mitochondrial quality control which allows defective mitochondria to be segregated from the pool of healthy mitochondria and removed by mitophagy [58].

Atg32 fulfills the role of a central receptor for multiple mitophagy-inducing pathways and determines the final turnover of mitochondria [26,27,59]. Therefore, a thorough regulation of its activity is anticipated. Besides regulation of its gene expression, posttranslational modification seems to control Atg32-mediated mitophagy [27,32,35,60]. However, the exact pathways of damage sensing and Atg32 activation are not fully characterized. Low expression level of Atg32 and its instability has prevented a thorough investigation of its posttranslational modification. In order to stabilize Atg32, we blocked mitochondrial degradation in the vacuole, by deleting the central protease Pep4. Surprisingly, deletion of *PEP4* did not stabilize Atg32. Instead, in the absence of the vacuolar protease Pep4, a high molecular weight form of Atg32 becomes stabilized. The size shift in gel electrophoresis corresponds to a covalent modification of 20 kDa. The modification is dependent on mitophagy induction and was found under conditions of rapamycin treatment, nitrogen starvation and mitophagy induction in post-log cells. As the modification accumulated within the first 15 min of rapamycin treatment we speculate that it is modulating the receptor activity. The nature of the modifying moiety is yet unclear. We excluded any modification via disulfide bridges, as all samples have been treated with β-mercaptoethanol before analysis by SDS-PAGE. As a central receptor in a highly regulated process, Atg32 is known to be phosphorylated [32,35,60]. However, the observed change in the migration of Atg32 is not typical for phosphorylation, which usually induces only moderate reduction in protein mobility and the modification was not sensitive to alkaline phoaphatase treatment. We also showed that treatment with the deubiquitinating enzyme USP2 had no influence on the modification. As the IMS domain is not required for mitophagy, and the autophagic machinery primarily resides in the cytosol, we speculated that the modification occurs in the cytosolic domain of the protein [33]. We therefore used a construct, in which the cytosolic domain was deleted, and found that the modification does not occur on this construct. We therefore suggest that the cytosolic domain is essential for modification, however we cannot rule out that it participates in the modification only indirectly.

In order to learn more about the 20 kDa Atg32 modification, we tested the involvement of the core autophagic machinery [61,62]. Atg32 modification is unperturbed in the absence of the vacuolar lipase Atg15, indicating that the modification occurs before vacuolar fusion. The core autophagy machinery, including Atg8 and Atg11, was found to be essential for the modification. We therefore suggest that modification occurs during or after targeting selected mitochondria for autophagy. Very unexpectedly we found Atg14 to be dispensable for Atg32 modification. In the absence of Atg14 PAS recruitment of autophagy components, including Atg8, is disturbed [14,15]. It is unclear, if other PI3 Kinases can complement for the function of Atg14 in Atg32 modification.

With Atg32 not being degraded by vacuolar proteases, we assumed that other regulatory proteases might be involved in its stability. The proteasome is a major degradation pathway in the cell, which is known to be required for the degradation of membrane proteins in the endoplasmatic reticulum (ER) [63]. Evidence also exists for the involvement of the proteasome in the degradation of mitochondrial proteins (mitochondria-associated degradation, MAD) [64–66]. We therefore tested, if the proteasome is also involved in the degradation of Atg32. In proteasome-deficient yeast no accumulation of modified forms of Atg32 was observed. Moreover, we observed that the degradation of non-modified Atg32 continued. Together, these results suggest a yet unknown protease responsible for the turnover of unmodified Atg32.

Blocking the proteolytic pathways of Atg32 would give us the opportunity for affinity purification of the active receptor complex in order to characterize novel interaction partners. As a first step towards an affinity isolation protocol, we tested if Atg32 would be sufficiently stabilized for mitochondrial isolation in a *Pep4-*deficient yeast strain. Indeed, an optimized cellular fractionation protocol revealed modified Atg32 on mitochondria in *Pep4*-deficient yeast (S1 Fig). However, due to ongoing proteolysis, the procedure failed to sufficiently enrich Atg32 for affinity isolation. More comprehensive knowledge about the proteolytic pathways of Atg32 will be necessary to allow the stable isolation of the active Atg32 signaling complex and the analysis of its interaction partners. In this context it will be interesting, how the modification of Atg32 affects the interaction with known interaction partners like Atg8 or Atg11.

Different degradation pathways of non-modified Atg32 and modified Atg32 allow for the discrimination between healthy and damaged mitochondria (Fig 4D). During mitophagy induction Atg32 is activated by a posttranslational modification. The modification of Atg32 targets mitochondria for the degradation in the vacuole. A block in vacuolar proteolysis in the absence of Pep4 allowed us to stabilize the modified form of Atg32. On mitochondria, not selected for mitophagy, Atg32 is continuously degraded. Surprisingly, vacuolar degradation is not responsible for the protein stability, indicating that Atg32 is routed to a separate pathway of degradation. We were able to exclude the proteasome to be involved in degradation of Atg32 on healthy mitochondria. We therefore conclude that a yet unknown protease accounts for this process.

## Supporting Information

S1 Fig Cellular fractionation in cytosol and mitochondrial fraction and detection of modified Atg32. Antibodies against the cytosolic protein Pgk1 and the mitochondrial Tom40 were used as a control.
(EPS)
